# Supplementary material for: Oxygen evolution reaction dynamics monitored by an individual nanosheet-based electronic circuit
Source: Nat Commun. 2017 Sep 21;8:645. doi: 10.1038/s41467-017-00778-z (PMC5608767; doi:10.1038/s41467-017-00778-z)
Supplement: Supplementary file 1 — Supplementary Information [file 41467_2017_778_MOESM1_ESM.pdf]

**Description of Supplementary Files**

File Name: Supplementary Information
Description: Supplementary Figures, Supplementary Tables, Supplementary Notes and Supplementary References

File Name: Peer Review File

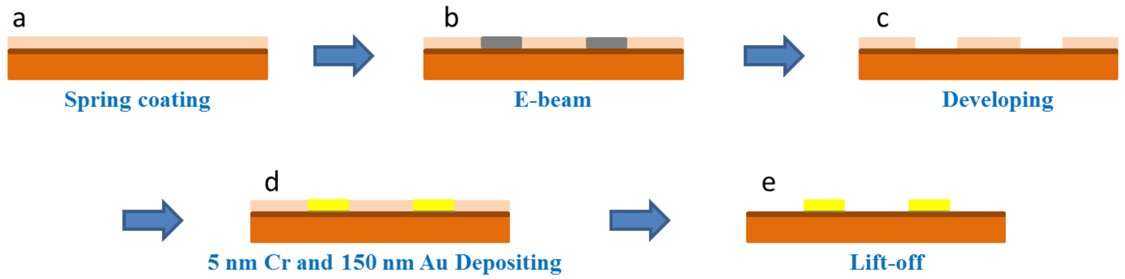


**Supplementary Figure 1.** A schematic illustration of the metallic electrodes fabrication process. This process includes the following steps: Step a. MMA and PMMA photoresists are coated on silicon wafers with 300 nm $SiO_2$ layer via spin-coating method; Step b. patterning the metal contacts pad with EBL on silicon wafer which is covered by MMA/PMMA photoresists; Step c. developing to remove the target photoresists which was patterned by EBL in step b; Step d. depositing Cr and Au on to the above silicon wafers with thermal evaporation; Step e. lifting off and obtaining target metallic patterns as metallic electrodes in devices.

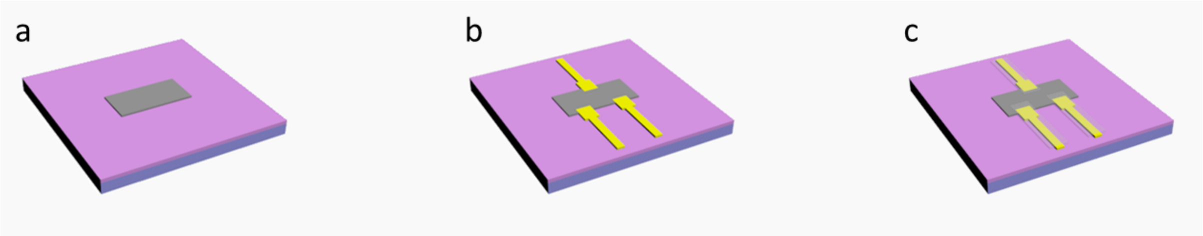


**Supplementary Figure 2.** Schematic illustration of the anode fabrication process. Three main steps are included：a. depositing prepared nanosheets onto Si wavier with 300 nm $SiO_2$ layer via spin-coating method; b. padding metallic electrodes to contact with target nanosheets; c. coating SU-8 2002, as a passivation layer, to cover the metallic electrodes, avoiding leakage current which transfers from metal contact to electrolyte.

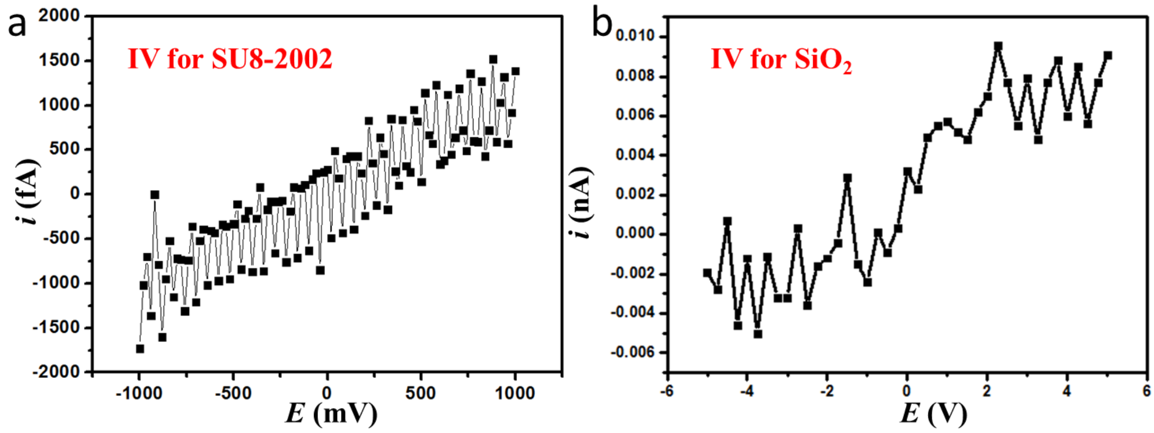


**Supplementary Figure 3.** Resistance of different parts within micro-device. a. The I-V curve of SU8-2002; b. The I-V curve of $SiO_2$.

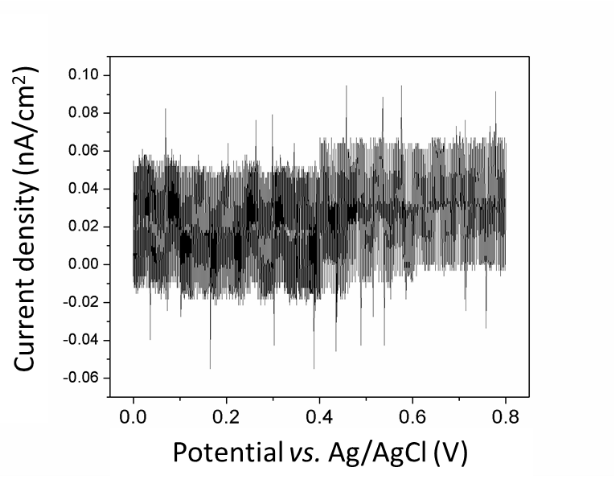


**Supplementary Figure 4.** CV curve of a comparable nanodevice sample.

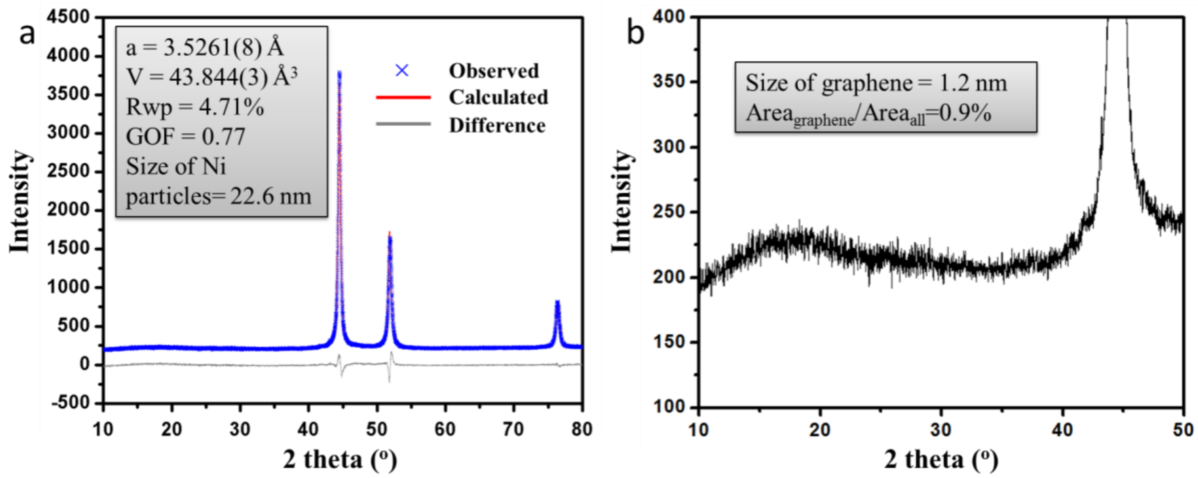


**Supplementary Figure 5.** A RIETVELD refinement of XRD. a. Ni particles; b. graphene.

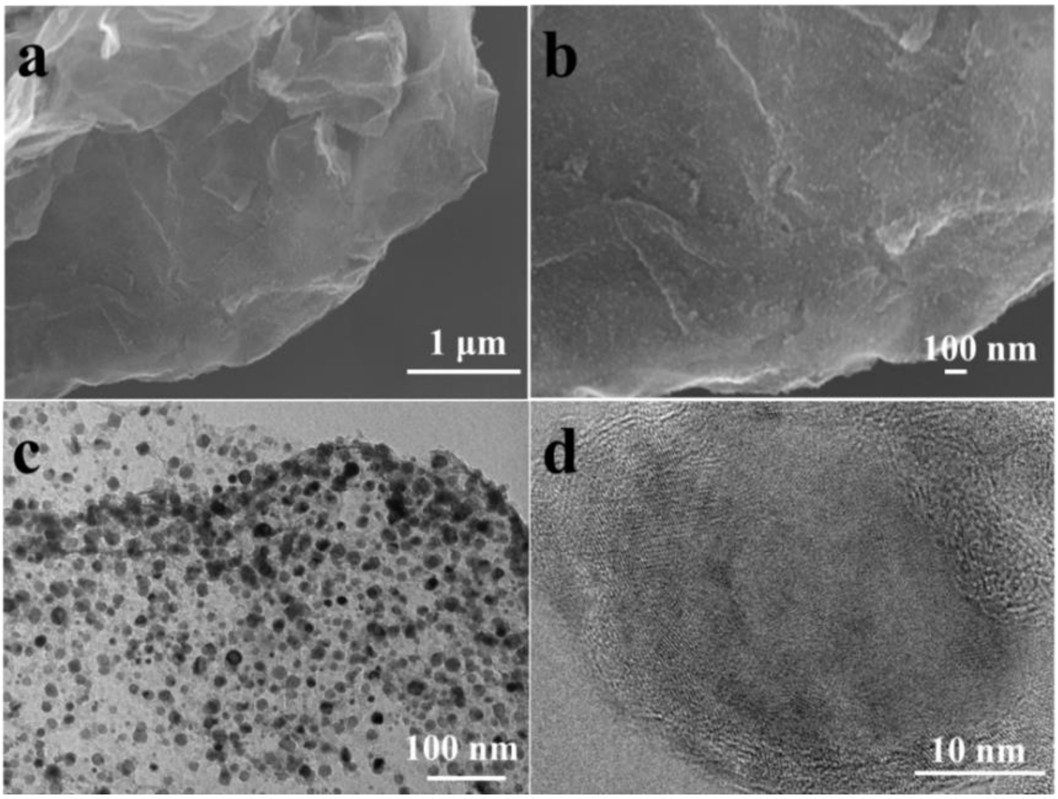


**Supplementary Figure 6.** Representative SEM (a, b) and TEM (c, d) images of as-prepared Ni-Graphene nanosheets on the device.

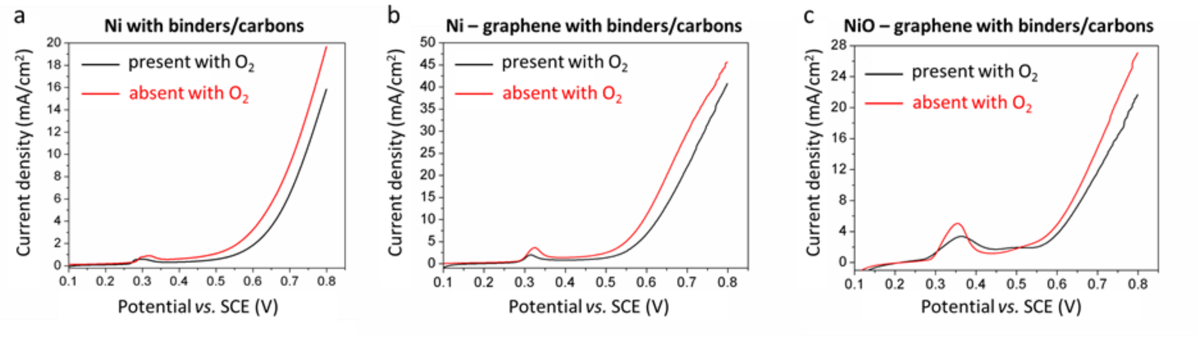


**Supplementary Figure 7.** OER activity of bulk catalysts under conventional measurement. a. Oxygen evolution currents of Ni catalyst measured in 1M KOH. b. Oxygen evolution currents of Ni-graphene as the catalyst measured in 1M KOH. c. Oxygen evolution currents of NiO-graphene as the catalyst measured in 1M KOH.

At a scan rate of 5 mV/s, for Ni catalyst (Supplementary Fig. 7a), a current density of 10 $mA/cm^2$ was achieved at a potential of 0.71 V vs. SCE under oxygen-absence condition, which is lower than that under the oxygen-presence condition, i.e., 0.742 V vs. SCE. In the case of Ni-graphene (Supplementary Fig. 7b), a current density of 10 $mA/cm^2$ occurs at 0.593 V vs. SCE under oxygen-absence condition, whereas a higher potential of 0.627 V vs. SCE can be seen under oxygen-presence condition. A similar trend is also observed in the NiO-graphene catalysts (Supplementary Fig. 7c). Results are consistent to our new setup.

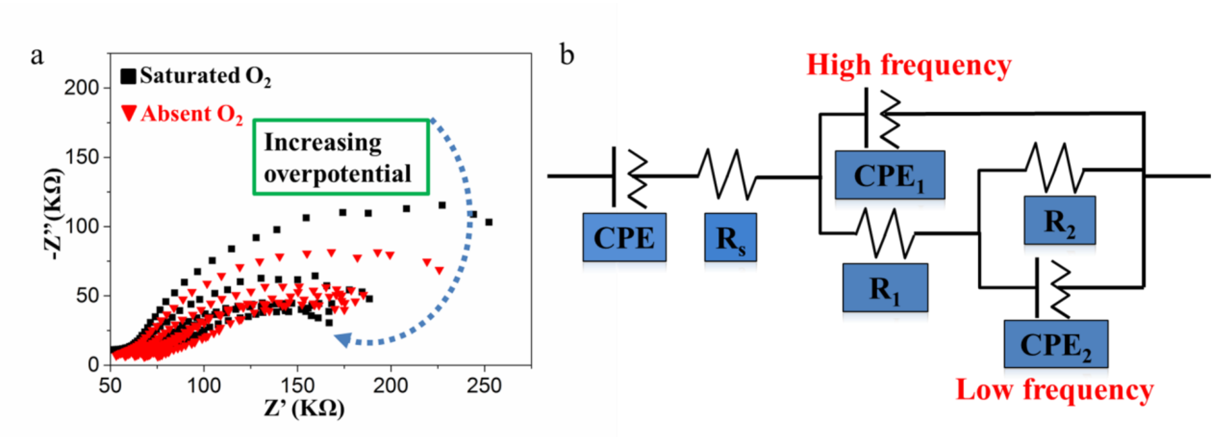


**Supplementary Figure 8.** a. Nyquist plots of the impedance data for Ni-Graphene nanosheet on device. b. The equivalent circuit used to fit the data.

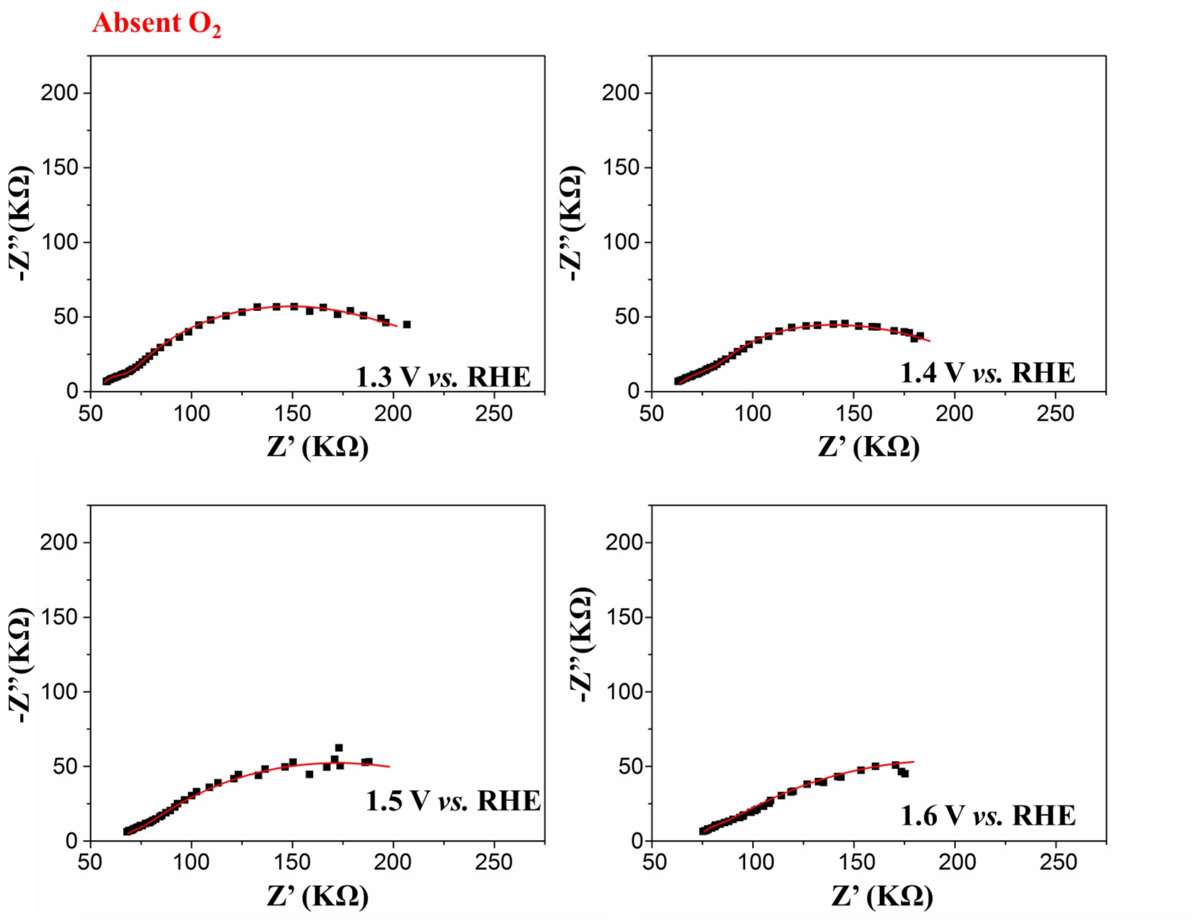


**Supplementary Figure 9.** The individual EIS plots in the DC potential range from 1.3 V to 1.6 V vs. RHE together with corresponding fitted curve based on the equivalent circuit model in Supplementary Fig. 8b for absent $O_2$ environment.

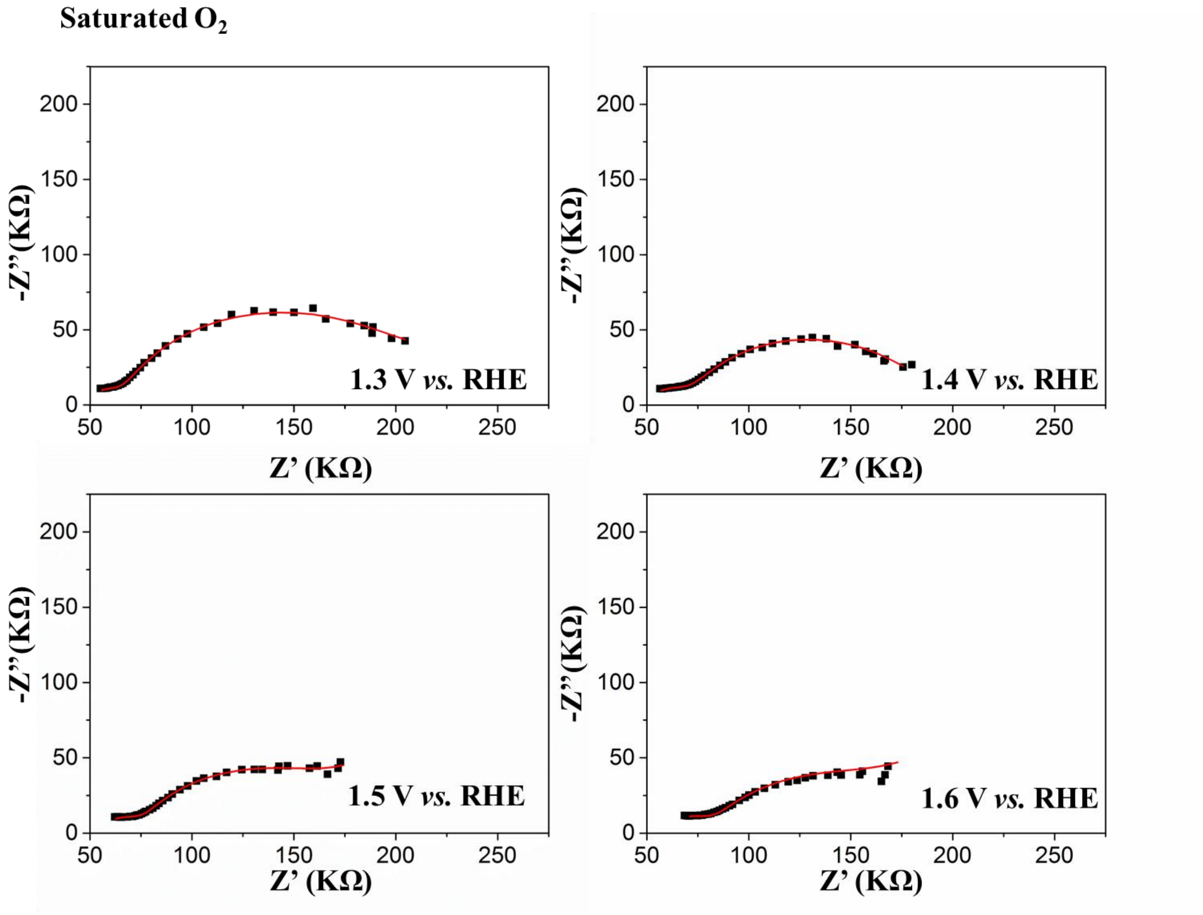


**Supplementary Figure 10.** The individual EIS plots in the DC potential range from 1.3 V to 1.6 V vs. RHE together with corresponding fitted curve based on the equivalent circuit model in Supplementary Fig. 8b for saturated $O_2$ environment.

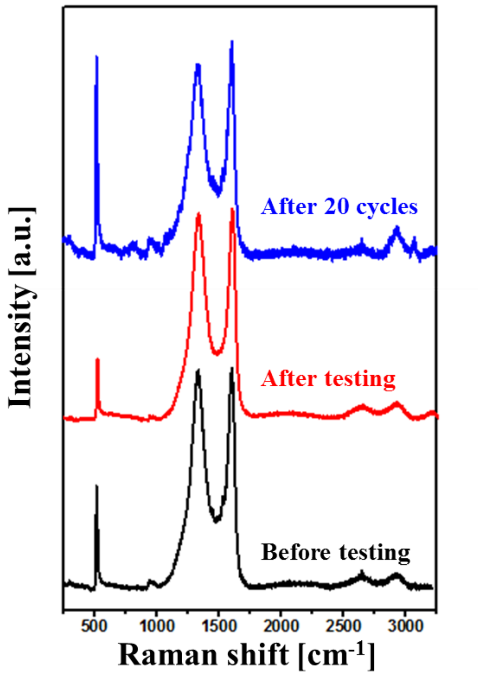


**Supplementary Figure 11.** Raman spectra of Ni-Graphene nanosheet before (0 cycles) and after OER testing (1 and 20 cycles).

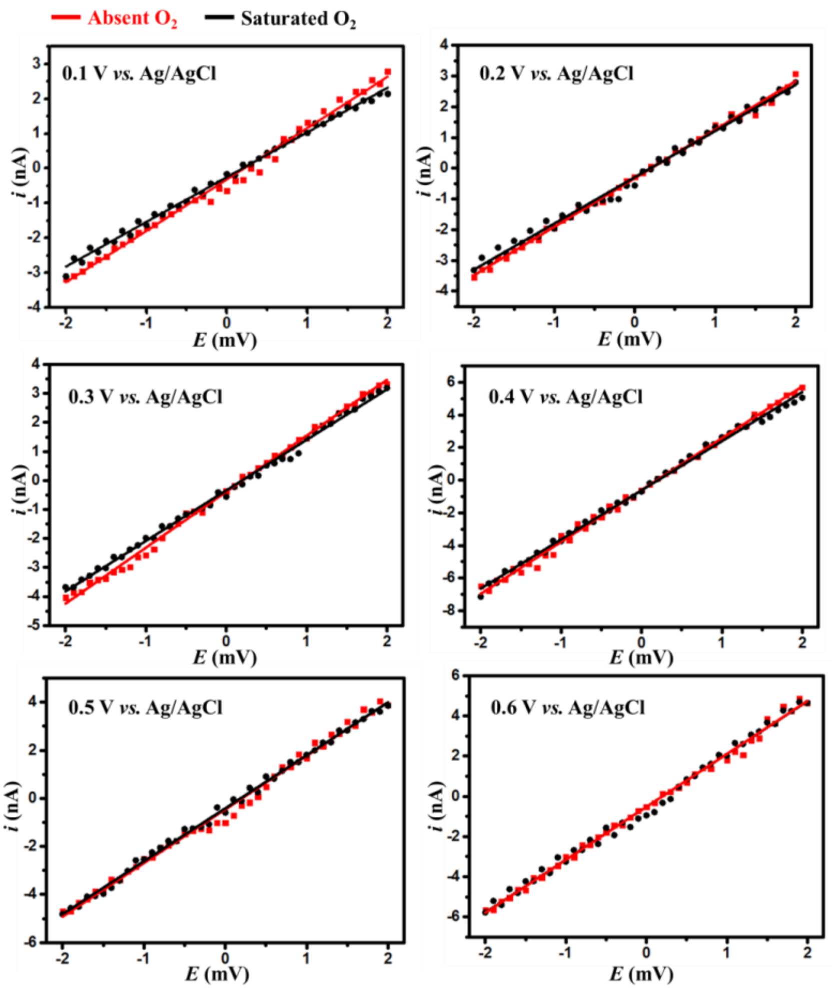


**Supplementary Figure 12.** The individual IV curves in the DC potential range from 0.1 V to 0.6 V vs. Ag/AgCl together with corresponding fitted curve.

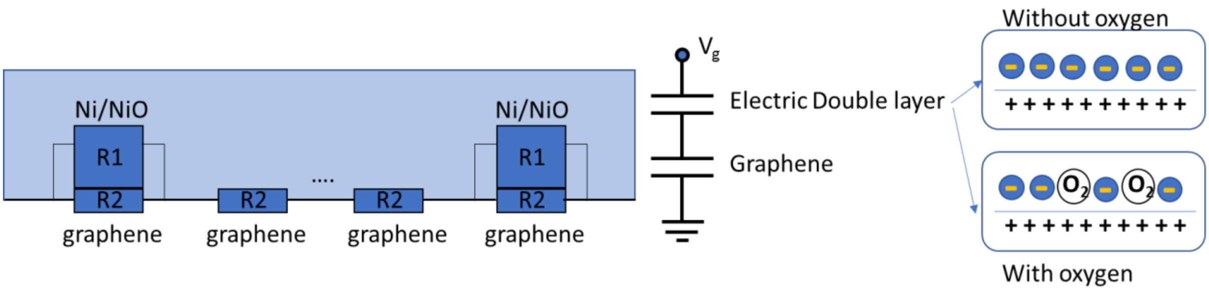


$$\frac{V_{EDL}}{V_{matrial}} \propto \frac{C_{material}}{C_{EDL}}$$

**Supplementary Figure 13.** Schematic diagram of electrochemical gate tuned nano-device.

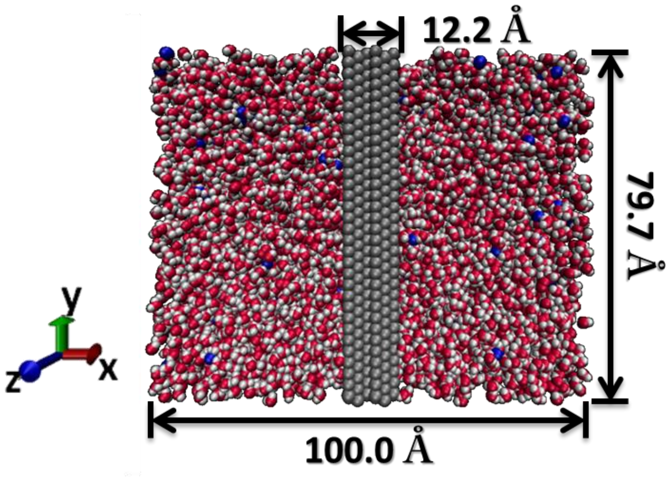


**Supplementary Figure 14**. A typical system in our MD simulations.

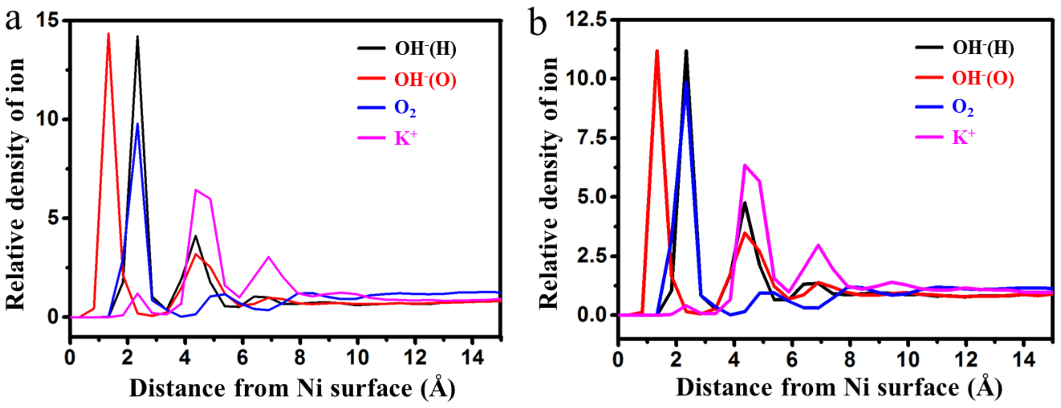


**Supplementary Figure 15.** a. Relative number density ρ of different electrolyte ions as a function of distance from the Ni cathode surface with oxygen concentration of 0.24 mmol/cm$^3$. b. Relative number density ρ of different electrolyte ions as a function of distance from the Ni cathode surface with oxygen concentration of 0.48 mmol/cm$^3$.

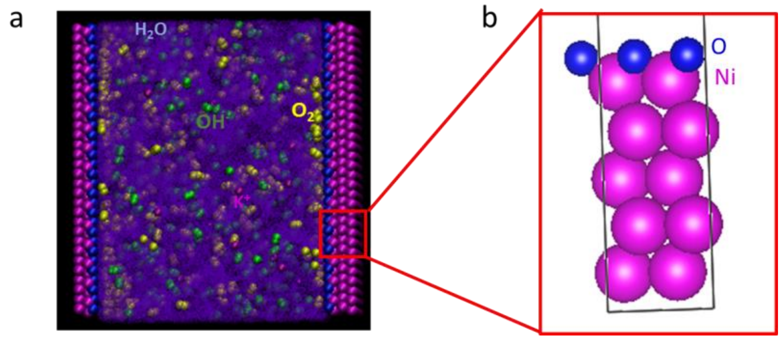


**Supplementary Figure 16.** a. A typical system in NiO-Ni MD simulations. b. A representative unit cell structure of NiO-Ni.

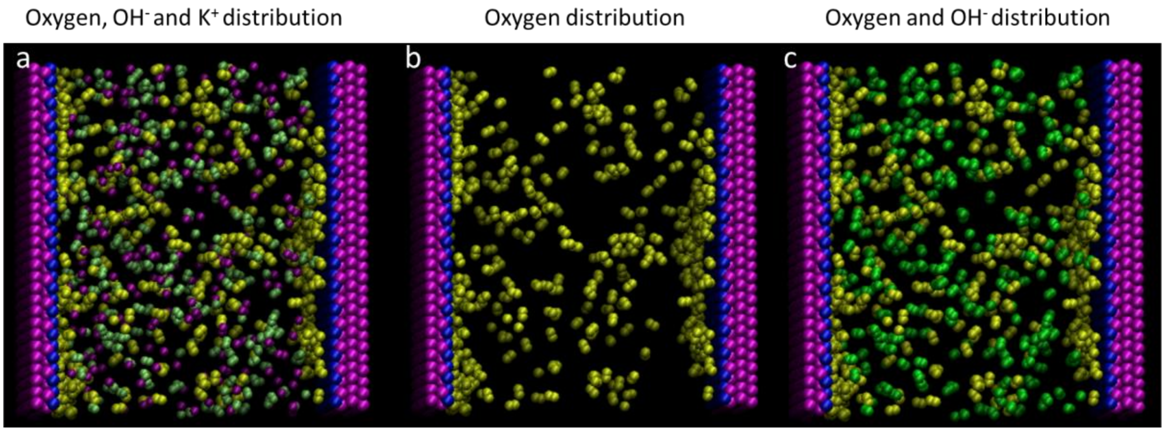


**Supplementary Figure 17.** Ion distribution in the solution. a. oxygen, $OH^-$ and $K^+$ distribution. b. oxygen distribution. c. oxygen and $OH^-$ distribution.

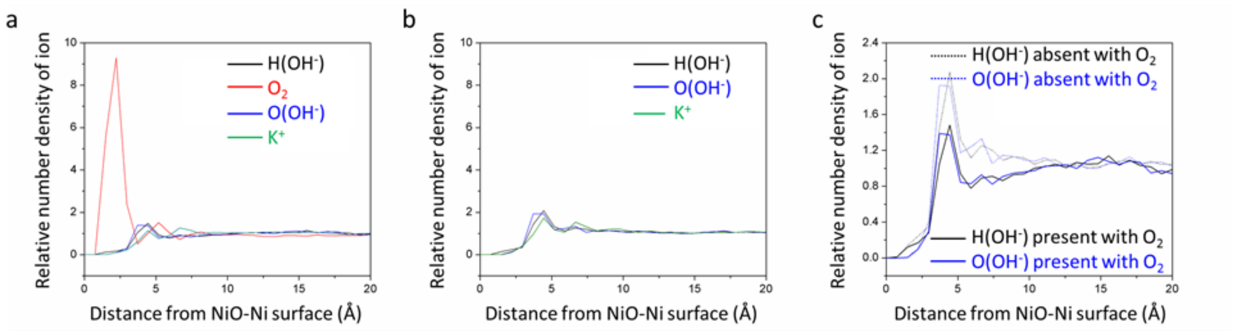


**Supplementary Figure 18.** MD results. a. Relative number density of atoms as a function of distance from NiO-Ni surface under oxygen-presence condition. b. Relative number density of atoms as a function of distance from NiO-Ni surface under oxygen-absence condition. c. A comparison of $OH^-$ relative number density distribution between the oxygen-presence and oxygen-absence conditions.

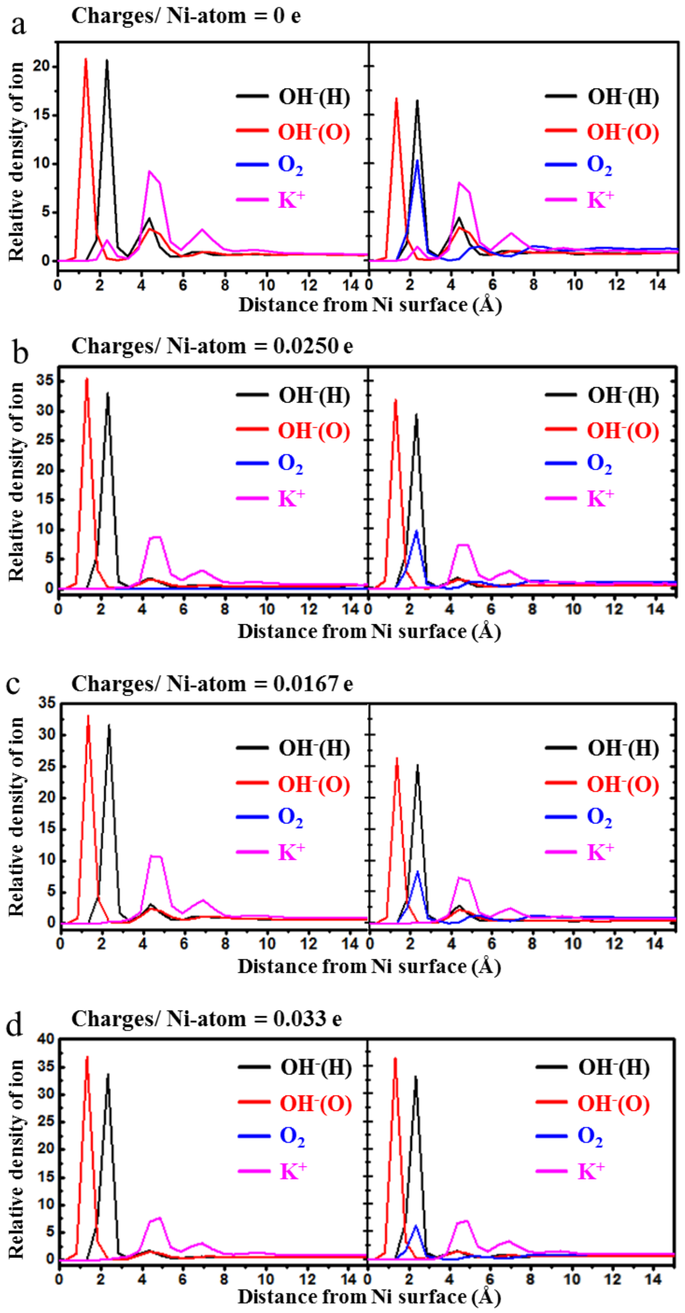


**Supplementary Figure 19.** a, b, c, and d. Relative number density ρ of different electrolyte ions as a function of distance from the Ni cathode surface with a charge of 0, 0.0250, 0.0167, 0.033 e/Ni-atom without oxygen in electrolyte (left) compared with the oxygen concentration of 0.12 mmol/cm$^3$ (right).

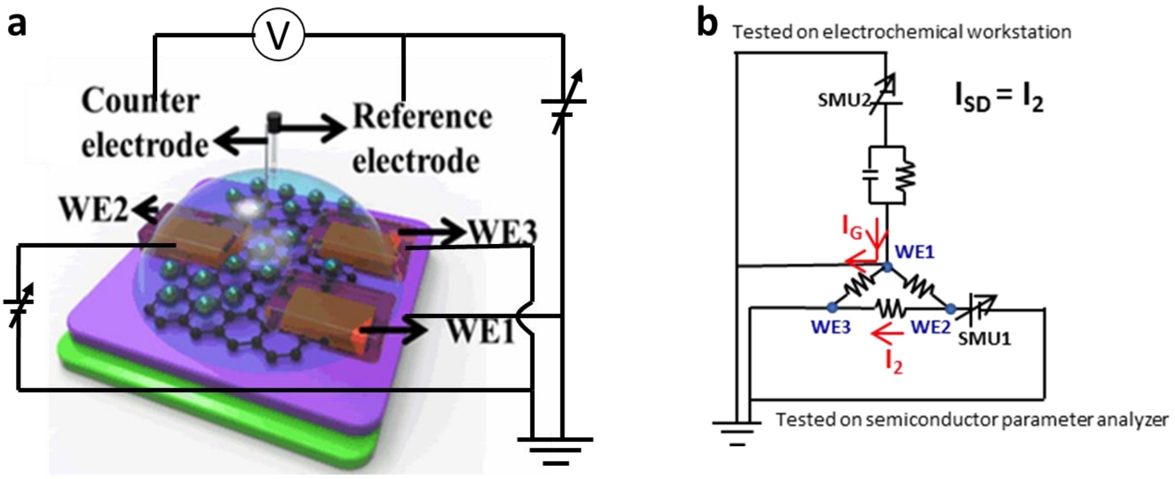


**Supplementary Figure 20.** On-chip electrical transport spectroscopy comparison (ETS). a. schematic illustration of the concurrent measurements of electrochemical (Ig) and electrical transport (Isd) characteristics of this work. b. Equivalent circuit model of the ETS (gate) measurement in this work.

**Supplementary Table 1:** Parameters of the Lennard-Jones force fields that were used in this paper

| Atoms | ε(eV) | σ (Å) | *M*(g/mol) | Valence(e) |
|---|---|---|---|---|
| Ni | 0.51962 | 2.282 | 58.693 | 0 ~ +0.033 |
| H(OH-) | 0.00000 | 0.000 | 1.0080 | +0.205 |
| O(OH-) | 0.00660 | 3.154 | 15.999 | -1.205 |
| K+ | 0.00377 | 3.143 | 39.098 | +1 |
| O(TIP3P) | 0.00660 | 3.151 | 15.999 | -0.834 |
| H(TIP3P) | 0.00000 | 0.000 | 1.0080 | +0.417 |
| O(O2) | 0.00520 | 3.029 | 15.999 | 0 |

**Supplementary Table 2:** Number of water molecules and electrolyte ions in one MD simulation super cell under different charging conditions

| Total charge on one Ni supercell surface (2,400 atoms) | K+ | OH- | water |
|---|---|---|---|
| 0 e | 340 | 340 | 18820 |
| +20 e | 330 | 350 | 18820 |
| +40 e | 320 | 360 | 18820 |
| +60 e | 310 | 270 | 18820 |
| +80 e | 300 | 380 | 18820 |

**Supplementary Table 3:** Parameters of the Lennard-Jones force fields for the interaction between several specific atoms using mixing rule

| | ε | σ |
|---|---|---|
| $O(O_2)$ - Ni | 0.052 | 2.66 |
| $O(OH^-)$ - Ni | 0.059 | 2.72 |
| $H(OH^-)$-Ni | 0 | 0 |
| $O(O_2)$ - Ni(NiO) | 0.052 | 2.66 |
| $O(O_2)$ - O(NiO) | 0.006 | 3.29 |
| $O(OH^-)$ - Ni(NiO) | 0.059 | 2.72 |
| $H(OH^-)$-Ni(NiO) | 0 | 0 |
| $O(OH^-)$ - O(NiO) | 0.007 | 3.35 |
| $H(OH^-)$-O(NiO) | 0 | 0 |

**Supplementary Note 1.** Fabrication of the Ni-Graphene nanosheet for electrochemical nanodevice

An n++ silicon (Si) wafer with 300 nm thermal oxide was used as device substrate. The fabrication process of Ni-Graphene nanosheet based device is schematically demonstrated in Supplementary Fig. 2. Typically, the pre-prepared free standing Ni-Graphene nonasheets were firstly deposited onto the substrate surface. A poly methyl methacrylate (PMMA Resist 950 A4 MicroChem Corp.) and a copolymer methyl methacrylate (MMA(8.5) MAA EL11 MicroChem Crop.) films were prepared respectively by spin-coating (500 r/min for 10s followed with 4000 r/min for 40s) on the substrate surface with pre-patterned metallic electrodes (Cr/Au, 5 nm/150 nm). E-beam lithography (EBL) was then used to open windows on MMA/PMMA, which created desired patterns on the substrate. Next, metallic films discussed above were deposited on it via a technology of physical vapor deposition (PVD) and obtained desired electrodes contacting with Ni-Graphene nanosheet after lifting off. To eliminate the influence of electrolyte and to avoid electrochemical reactions on the metal electrodes, another layer of SU8-2002 was then deposited on the nonosheet device with spin-coating, through EBL and lifting off, allowing all metallic electrodes to be covered by SU8-2002 layer. The final device, with exposed nanosheet and SU8-2002 protected electrodes was used for in-device electrochemistry and electrical spectroscopy.

**Supplementary Note 2.** Synthesis of Ni-Graphene nanosheets

In a typical synthesis, 0.546 g $Ni(NO)_3$•$6H_2O$, 0.616 g 2-methyl imidazole, 0.1 g polyvinyl pyrrolidone and 5 mL graphene oxide (1.5 g/L) were added in 30 mL methanol and mixed by stirring and then transferred into a telfon-lined stainless steel autoclave and kept at 140 ℃ for 10 h. The precursor was collected and washed repeatedly with ethanol, and finally dried at 80 ℃ for 12 h in air. Finally, after the precursor was sintered at 550 ℃ for 3 h, Ni particles on graphene were obtained.

**Supplementary Note 3.** Electrochemical characterization

X-ray diffraction measurement (XRD) was performed to investigate the crystallographic information of Ni-Graphene materials using a D8 Discover X-ray diffractometer with a non-monochromated Cu Kα X-ray source (k = 1.5406 Å). Field-emission scanning electron microscopic (SEM) images were collected with a JSM-7001F instrument at an acceleration voltage of 10 kV and the transmission electron microscopic (TEM) images and Raman spectrum were recorded with the JEM-2100F STEM/EDS and RENISHAW.

**Supplementary Note 4.** Electrochemical impedance spectroscopy (EIS)

This model is characterized by one high frequency time constant ($\tau 1$, CPE1-R1) and low frequency time constant ($\tau 2$, CPE2-R2), both which changes with overpotential as illustrated by a decrease of diameter of both semi-circles with increasing overpotential neither with absent $O_2$ or saturated with $O_2$.

The experimental impedance data were fitted to the equivalent circuit model with a hundred of iterations in NOVA 1.10, an electrochemical work station software. The EIS data in the DC potential range between 1.2 V and 1.6 V vs. RHE together with the corresponding fitted curve are displayed in and the equivalent circuit model is seen to the EIS data very well.

**Supplementary Note 5.** MD simulation

To explore the influence of oxygen molecules on the ion concentration near the surface of active materials (Ni), we carry out molecular dynamics (MD) simulations of sodium hydroxide (KOH) solution on Ni surface. Specifically, water molecules were filled in the supercell to generate a pressure close to 1 bar at 300 K. The numbers of the electrolyte ions were determined with two conditions: the solution concentration (set as 1 mol/L) and charge neutrality of the whole system. In our simulations, different charge conditions for active materials (Ni) were investigated. Given the surface capacitance value of Ni was 20 uF/$cm^2$ and electric potential window as 1 V[1], the maximum charge per target Ni atom (upper Ni atoms which are exposed to electrolyte) is calculated to be 0.033e. Accordingly, 0, +0.0083, +0.0167, +0.025 and +0.033e was imposed on each target Ni atom respectively to consider different charging conditions. Supplementary Table 1 summarizes the numbers of water molecules and ions in the simulation systems.

The crystal structure of Ni is the face-centered cubic lattice with lattice constant of 3.524 Å, and the (1, 1, 1) surface of the crystal lattice is exposed to the electrolyte by defining the x-y-z orientations to be (1, 1, 1) (1, -1, 0) (1 1 -2) respectively. The TIP/3P model was used for water. The interactions among Ni, water, KOH, and oxygen molecules ($O_2$) were described by Lennard-Jones (LJ) pairwise potential[2]. The potential function has the form of:

$$\mathrm{E}(\boldsymbol{r_{ij}}) = 4\boldsymbol{\varepsilon_{ij}}\left[\left(\frac{\boldsymbol{\sigma_{ij}}}{\boldsymbol{r_{ij}}}\right)^{\mathbf{12}} - \left(\frac{\boldsymbol{\sigma_{ij}}}{\boldsymbol{r_{ij}}}\right)^{\mathbf{6}}\right]$$

where, $r_{ij}$ denotes the distance between atoms, $\varepsilon$ characterizes the strength of the interaction and $\sigma$ determines the distance at which the two atoms are at equilibrium. The substituted *i* and *j* denote the component atoms and ions of the whole model. Parameters of LJ potentials of $K^+$ ions, $O_2$ molecules and Ni atoms were taken from CHARMM force field[3], expect that of $OH^-$

ions taken from reference 4. Supplementary Table 2 summarizes the parameters and the charges of each type of ions.

All MD simulations were conducted using LAMMPS package. Here, the positions of Ni atoms were frozen. The Berendsen thermostat was adopted to control system temperature. The NVT ensemble simulations at 298 K were carried out for 2.5 ns to ensure that the systems indeed reached thermal equilibrium state. Results of last 1 ns were taken for the concentration profile analyze.

**Supplementary Note 6.** In-device electrochemical characterization

The Ag/AgCl/saturated KCl reference electrode was calibrated with respect to reversible hydrogen electrode (RHE). The calibration was performed in the high purity hydrogen saturated electrolyte with a Pt foil as the working electrode. CVs were run at a scan rate of 1 mV $s^{-1}$, and the average of the two potentials at which the current crossed zero was taken to be the thermodynamic potential for the hydrogen electrode reactions. This calibration resulted in a shift of +0.95 V versus the RHE.

**Supplementary References:**


1. Shervedani, R. K. & Lasia, A. Evaluation of the surface roughness of microporous Ni–Zn–P electrodes by in situ methods. *J. Appl. Electrochem.* **29**, 979-986 (1999).

2. MacKerell Jr, A. D. *et al.* All-atom empirical potential for molecular modeling and dynamics studies of proteins. *J. Phys. Chem. B* **102**, 3586-3616 (1998).

3. Foloppe, N. & MacKerell Jr, A. D. All‐atom empirical force field for nucleic acids: I. Parameter optimization based on small molecule and condensed phase macromolecular target data. *J. Comput. Chem.* **21**, 86-104 (2000).
